# Inactivation of *Bacillus* *anthracis* Spores

**DOI:** 10.3201/eid0906.020377

**Published:** 2003-06

**Authors:** Ellen A. Spotts Whitney, Mark E. Beatty, Thomas H. Taylor, Robbin Weyant, Jeremy Sobel, Matthew J. Arduino, David A. Ashford

**Affiliations:** * Centers for Disease Control and Prevention, Atlanta, Georgia, USA

**Keywords:** Bacillus anthracis, sporicidal efficacy, heat inactivation, chemical sterilization, gaseous sterilization, and radiation, synopsis

## Abstract

After the intentional release of *Bacillus anthracis* through the U.S. Postal Service in the fall of 2001, many environments were contaminated with *B. anthracis* spores, and frequent inquiries were made regarding the science of destroying these spores. We conducted a survey of the literature that had potential application to the inactivation of *B. anthracis* spores. This article provides a tabular summary of the results.

In October 2001, several letters containing *Bacillus anthracis* spores were sent through the U.S. Postal Service to recipients in government and private-sector buildings. Consequently, 23 human inhalational or cutaneous anthrax infections occurred. Five of the 11 inhalational anthrax infections were fatal (*1*,*2*). As a result of this intentional release of *B. anthracis*, several post offices, mailrooms in government buildings, and private office buildings were contaminated with *B. anthracis* spores. During the initial response, frequent requests were made for published materials about inactivating *Bacillus* spores. However, no adequate single source of literature on this subject was available. Because of the risk to humans, remediation of anthrax-contaminated buildings and their contents has been the focus both of scientific discussion and commercial product marketing. A number of manufacturers have developed equipment or materials that reportedly kill *B. anthracis* spores. However, these manufacturers have tested their products with laboratory tests that use *Bacillus* species other than *B. anthracis*, and the efficacy of some of these technologies relies on published literature. An obvious concern is whether postremediation levels of spores are safe; the summarized studies make no claim about whether a safe level exists and what it might be.

We provide a summary of much of the available literature on the inactivation of *Bacillus* spores that is relevant to the inactivation of *B. anthracis*. We reviewed publications from 1930 to 2002, and we have created a tabular summary of those articles. Treatments or agents commonly cited include heat, formaldehyde, hypochlorite solutions, chlorine dioxide, and radiation. Methods regarding inoculum size, concentration, and other variables are not consistent between experiments, but each experiment provides some specific information of value. Early studies that lack quantitative data are not included. A number of the cited studies address *Bacillus* species other than *B. anthracis*. We include these for information, with the caveat that surrogates do not always predict the behavior of the target species. Furthermore, the results from laboratory experiments do not specifically address questions regarding the best methods for inactivating spores on different materials such as mail, carpet, other porous objects, food, or water. Transfer of these sporicidal methods from the laboratory to a building has not yet been tested; however, the known laboratory results are a logical place to start when considering the decontamination of a building.

Decontamination is defined as the irreversible inactivation of infectious agents so that an area is rendered safe. However, decontamination may not eliminate bacterial spores. Sterilization is the complete destruction or elimination of microbial viability, including spores (*3*).

The experiments described provide a logical starting point for future experiments and decontamination strategies in the event of anthrax bioterrorism. Our intent is not to provide a comparative evaluation or recommendations for decontamination but rather to summarize the quantitative published results and provide a useful reference.

## Review

Variations in time, temperature, concentration, pH, and relative humidity may affect the sporicidal activity of various agents. Accordingly, and especially for real-world situations, attention must be paid simultaneously to more than one controllable or uncontrollable factor. In Tables 1 and 2 and in the discussion, we address some of the key ancillary factors.

**Table 1:** Heat inactivation of *B. anthracis* spores^a^

Temperature	Time	Inoculum size	Inactivation effect	Ref.
**Boiling**				
100°C	10 min	3 x 10^6^	Sample sterilized	4,5
	5 min	7.5 x 10^8^	Sample sterilized	
**Moist heat**				
90°C	20 min	1.2 x 10^6^	Sample sterilized	4,5
90°C to 91°C	60 min	3 x 10^8^	Spores detected	
100°C	10 min	1.2 x 10^6^	Sample sterilized	5,6
100°C to 101°C	17 min	1 x 10^5^	Sample sterilized	
105°C	10 min	3 x 10^6^	Sample sterilized	5
120°C	15 min	2.4 x 10^8^	Sample sterilized	4
**Dry heat**				
140°C	>90 min	6 x 10^3^ to 1.2 x 10^4^	Sample sterilized	7
150°C	10 min	6 x 10^3^ to 1.2 x 10^4^	Sample sterilized	
160°C	10 min	6 x 10^3^ to 1.2 x 10^4^	Sample sterilized	
180°C	2 min	6 x 10^3^ to 1.2 x 10^4^	Sample sterilized	
190°C	1 min	6 x 10^3^ to 1.2 x 10^4^	Sample sterilized	
200°C	30 sec	6 x 10^3^ to 1.2 x 10^4^	Sample sterilized	

^a^Spores in liquid suspension exposed to flowing steam at 100°C.

**Table 2:** Efficiency of chemicals, gases, and radiation on the inactivation of *Bacillus* spores^a^

Method	Concentration		Inoculum size	Time			Efficiency	Ref.	
**Chemical sterilization**									
Calcium hypochlorite	20 ppm available; Cl_2_, pH 8.0, 20ºC		3 x 10^5^–4 x 10^5^ spores of *Bacillus subtilis* in 5.0 mL sterile distilled H_2_O	4.8 min			99% killed	8	
	25 ppm available; Cl_2_, pH 6.0, 20ºC		2 x 10^7^ spores/mL of *B. metiens* in 10 mL of sterile distilled H_2_O	2.5 min			0.061 (log of average % survivors) 99% killed	9	
Free available chlorine	2.4–2.3 mg/L available; CL_2_, pH 7.2, 22ºC		1.1 x 10^5^ spore suspension of *B. anthracis*	1 h			>99.99% killed (1 spore/mL survived)	10	
Sodium hypochlorite (NaOCl)	0.05%, pH 7.0, 20°C		Spore suspension of *B. subtilis* *globigii,* representing 1.6–2.2 x 10^9^ CFU/mL	30 min			99.99% killed	11	
	0.05%, pH 11.0, 20°C						50% spores survived		
Hydrogen peroxide (H_2_O_2_)	25.8%, 24°C		*B. subtilis globigii* spore suspension (no concentration)	15 min			0.001% survived	12	
	25.8%, 76°C			<1 min			<0.0001% survived		
	0.88 mol/L, pH 5.0		10^6^ CFU/mL *B. subtilis* spore suspension	3 h			100% killed	13	
	0.88 mol/L, pH 4.3		10 mL *B. subtilis* spore suspension coated onto stainless steel carriers	6 h			100% killed		
Peracetic acid (CH_3_COOOH)	0.13 mol/L, pH 5.0, 6.5, 8.0		10^6^ CFU/mL *B. subtilis*	<30 min			100% killed	13	
	0.39 mol/L, pH 4.0, 7.0, 9.0		10 mL *B. subtilis* spore suspension coated on stainless steel carriers	24 h			100% killed		
Formaldehyde (CH_2_O)		4% in water	10^8^/mL *B. anthracis*		2 h		10^4^ inactivation factor	14	
		400 mg/m^3^, 30% RH	10^2^–3 x 10^8^ *B. globigii* NCTC 10073 dried on disks		22 min		1 log_10_ reduction, at 23.5°C–25°C	15	
		280 mg/m^3^, 50%RH			31 min				
		250 mg/m^3^, 80% RH			16 min				
		400 mg/m^3^, 98% RH			9 min				
Glutaraldehyde (C_5_H_8_O_2_)		2% in water, pH 8.0	10^8^/mL spores *B. anthracis*		15 min		10^4^ inactivation factor	14	
Sodium hydroxide (NaOH)		5%, 27.8ºC	7 x 10^9^ spores/mL *B. subtilis*		1.5 h		99% killed	16	
		5%, 21.1ºC			3.6 h		99% killed		
**Gaseous sterilization**									
Ethylene oxide (C_2_H_4_O)		Exposed to constant boiling HCL at 20°C for 30 min before exposure to ethylene oxide at room temperature	*B. globigii* and *B. anthracis* dried onto suture loop carriers (no concentration)		1 h	100% killed		17	
		500 mg/L, 30%–50% RH, 54.4°C	~10^6^ spores *B. globigii* on nonhygroscopic surfaces		30 min	4-log reduction		18	
			~10^6^ spores *B. globigii* on hygroscopic surfaces			6-log reduction			
Chlorine dioxide (ClO_2_)		40 mg/L, 60%–80% RH, 25°C–27ºC	1.4 x 10^6^/0.2 mL *B. subtilis* subsp. *Niger* dried on paper and aluminum foil strips		1 h	100% killed		19	
		30 mg/L, 80%–85% RH, 30ºC	10^6^ spores/biologic indicator; *B. subtilis* subsp. *Niger*		30 min	100% killed (estimated time to kill 90%, 4.4 min)		20	
		6–7 mg/L, 20%–40% RH, 23ºC	10^6^ spores/biologic indicator; *B. subtilis* subsp. *Niger*		30 min	10^1^ CFU/biologic indicator (estimated time to kill 90%, 4.2 min)		21	
		70%–75% RH for 0.5 before exposure, 23ºC			15 min	0 CFU/biologic indicator (estimated time to kill 90%, 1.6 min)			
Hydrogen peroxide (H_2_O_2_) plasma		0.208 mg/L, 1.5 Torr pretreatment for 10 min; 2.49 MHz, 150 W of pulsed plasma in a cycle of 0.5 ms plasma on, 1.0 ms plasma off	3.4 x 10^5^ *B. subtilis* subsp. *globgii* spores on paper disks and packaged in spun-bonded polyethylene		15 min	100% killed		22	
Methylene bromide (CH_3_Br)		3.4–3.9 g/L, room temperature in the presence of moisture	1 x 10^5^–5 x 10^7^ spores of *B. anthracis* dried on sterile filter paper strips		24 h	100% killed		23	
Peracetic acid vapor (CH_3_COOOH)		1 mg/L, 80% RH	6 x 10^5^ – 8x 10^5^ *B. subtilis* *niger* dried on filter-paper disks and glass squares		10 min	<1 spore remained on paper and glass		24	
		1 mg/L, 60% RH				2 spores remained on paper; 38 spores remained on glass			
		1 mg/L, 40% RH				24 spores remained on paper; 1,530 spores remained on glass			
Propylene oxide (C_3_H_6_O)		1250 mg/L, 86% RH, 36°C–38ºC	9.5 x 10^5^–1.1 x 10^6^ spores *B. subtilis* *niger* dried on filter paper		1.05 h	90% killed		25	
		1000 mg/L, 37°C	2.5 x 10^7^ spores *B. subtilis* *niger* in cereal flakes		3 h	3.7% survived			
Ozone (O_3_)		1.0 mg/L generated in water pH 3	1.8 x 10^5^ spores/mL *B. cerus*		5 min	<10^1^ CFU/mL survived		26	
		3.0 mg/L, preconditioned at 54% RH	10^8^–2 x 10^8^ *B. subtilis* dried on filter paper		1.5 h 95% RH	<0.001% survived		27	
			10^8^–2 x 10^8^ *B. cerus* dried on filter paper		1.5 h 95% RH	<0.001% survived			
		900 ppm, preconditioned at 65%–70% RH for 15 h	5 x 10^7^ spores/glass coupon		30 min 80% RH	10^0^ survived		28	
					60 min 70% RH	10^0^ survived			
**Radiation**									
UV		85% 2537A	*B. anthracis* (mixed spores and vegetative forms) in beef extract agar pH 7.4 (no concentration)		452 ergs/mm^2^	90% killed		29	
		4,800 μWs/cm^2^	0.1 mL of 10^8^ *B. anthracis* spore suspension dried on aluminum carriers		<96h	2.4 log reduction, unreliable results		30	
		450,000 μWs/cm^2^	0.1 mL of 10^8^ *B. anthracis* spore suspension dried on ceramic carriers		<96h	2.03 log reduction, unreliable results			
		52.8 x 10^6^ μWs/cm^2^	0.1 mL of 10^8^ *B. anthracis* spore suspension dried on wood carriers		30h	0.67 log reduction			
Gamma irradiation			10^6^ spores/mL *B. anthracis*		Dose of 1 x 10^6^ rad	100% killed		31	

^a^RH, relative humidity; conversions: 1 ppm = 1 mg/L; mol/L = gram molecular weight/L; 1 rad = 100 ergs/g; and 1 watt = 10^7^ ergs/s.

Boiling water for >10 minutes, for example, can reduce *B. anthracis* spore counts by at least 10^6^ (Table 1). Variations in time and temperature conditions required to reduce spore counts listed in Table 1 can be attributed to differences in experimental conditions, strains of *B. anthracis* tested, or inoculum size.

The U.S. Environmental Protection Agency indicates that use of sodium hypochlorite as a sporicide is applicable under an emergency exemption (Section 18: Crisis Exemption; Federal Insecticide, Fungicide, and Rodenticide Act); as such, sodium hypochlorite may be used under the conditions specified (*32*). Given these conditions, the sporicidal effectiveness of hypochlorite solutions depends on the concentration of free available chlorine and pH. Common household bleach (sodium hypochlorite) has a pH of 12 to prolong its shelf life. To achieve effective sporicidal activity, bleach must be diluted with water to increase the free available chlorine and acetic acid to change the pH of the solution to 7 (*11*). Organic matter may decrease the sporicidal efficiency of sodium hypochlorite (*33*).

Concentration, humidity, temperature, and carrier material affect gaseous sterilization of spores. Ethylene oxide penetrates into porous material (absorbed strongly by rubber and many plastics); thus vapors are not readily eliminated by brief aeration. Ethylene oxide is also flammable (*34*). Residual spores were not completely killed after a 30-minute exposure to chlorine dioxide at a relative humidity of 20% to 40%, whereas all spores were killed after a 15-minute exposure to chlorine dioxide with the addition of prehumidification at a relative humidity of 70% to 75% (*21*). Peracetic acid vapor does not penetrate well into porous surfaces and is flammable. The amount of contamination, level of cleanliness of surfaces, and relative humidity will contribute to peracetic acid vapor’s effectiveness as a sporicide (*24*). Organic matter may absorb and chemically react with propylene oxide, reducing its effectiveness. Organic matter may also provide physical protection from the oxide (*25*). The sporicidal property of ozone is affected by relative humidity: as relative humidity decreases, the time required for killing organisms increases (*27*).

## Discussion

Decontamination of buildings from intentional release of *B. anthracis* is a new problem, and no accumulated scientific knowledge exists on the subject. Two areas of prior scientific research may be relevant: food processing and laboratory decontamination. With modification based on further study, the technologies used in laboratories and food processing plants may be applied to buildings.

Direct information on killing *B. anthracis* spores in foods by cooking is scarce, and the complexity of food matrices precludes easy extrapolation of the laboratory data into nonfood matrices. However, information on inactivating spores of bacterial species more resistant to environmental conditions than *B. anthracis* can provide guidance. The spores of *Clostridium botulinum* are more resistant to heat inactivation than are *B. anthracis* spores (*4*). The commercial retort process of canning achieves a 12-log reduction of *C. botulinum* spores, and by extension, should achieve a similar killing rate for *B. anthracis* spores. Further research in this area is needed.

Historically, formaldehyde solution or gas has been used both as a disinfectant and chemical sterilant. Formaldehyde was used to disinfect as early as the late 1880s and is still used to reprocess hemodialyzers for reuse on the same patient and to decontaminate biologic safety cabinets and laboratories (*35*–*37*). Formaldehyde gas has been used for fumigation in the poultry industry and for disinfection of biologic safety cabinets and laboratories (*38*,*39*). Data from controlled experiments with *B. globigii* NCTC 10073 spores have demonstrated the effect of humidity on formaldehyde concentration (mg/m^3^) to obtain a >8-log reduction in viable spores (*15*).

Fumigation with formaldehyde vapor (18 mg/L–21 mg/L) has also been used to treat a textile mill contaminated with ­*B. anthracis* spores (*40*). In this instance, contamination was greatly reduced immediately after treatment and was undetectable 6 months later. However, the possible role of formaldehyde as a carcinogen has limited its use. Formaldehyde can be neutralized with ammonium bicarbonate after fumigation, reducing its carcinogenic properties.

Gamma radiation was used in the 1960s and 1970s to disinfect *B. anthracis*–contaminated imported bailed goat hair. A study by Horne et al. suggested that a dose of 1.5 megarads from a 200,000-rad/hour cobalt source was sufficient to kill most resistant spores when mixed with goat hair; 2 megarads was recommended to include a margin of safety (*31*). After the intentional release of *B. anthracis* through the postal system in 2001, pursuing a decontamination method for the undelivered mail was essential. Gamma radiation was used to decontaminate all mail from contaminated facilities on the basis of these data.

## Summary

Multiple technologies may be needed to decontaminate buildings and their contents. As in a laboratory, where some items are wiped, some items are autoclaved, and some spaces are treated with gas, more than one method may be required for decontamination. Also, for certain decontamination tasks, e.g., cleaning small heat-proof and water-proof objects, more than one option will be available. Further, even within the context of one type of application (e.g., walls; ducts for heating, ventilating, air conditioning, and refrigeration; carpet; and small objects), potentially conflicting priorities exist between bioefficacy, logistics, and safety.

Our review suggests two conclusions. First, additional scientific research is needed. Although transferring the methods used to decontaminate or sterilize laboratory or food industry settings to decontaminating buildings may be useful, this transfer of methods has not been scientifically tested. Also, much of the data available is based on other *Bacillus* species; more testing with or correlation to *B. anthracis* contamination is suggested. Second, choosing between technologies is a complex issue, and a formal decision process would be useful. Various parties in the public and private sector have suggested numerous, sometimes disparate, methods for the inactivation of *B. anthracis* spores in contaminated environments. Further research is needed regarding improved methods for remediation of environments contaminated with *B. anthracis* spores, and the literature summarized here provides a basis for that effort.
